# The association of maternal education with access to maternal care and child mortality in Nigeria: A secondary data regression-based analysis

**DOI:** 10.1371/journal.pone.0337367

**Published:** 2026-02-05

**Authors:** Chekwube Madichie, James Lomas, Luigi Siciliani

**Affiliations:** 1 Department of Economics and Related Studies, University of York, York, United Kingdom; 2 Department of Economics and Related Studies, University of York, York, United Kingdom; 3 Department of Economics and Related Studies, University of York, York, United Kingdom; Federal Medical Centre Birnin Kudu, NIGERIA

## Abstract

**Background:**

Reducing under-five mortality and ensuring universal access to maternal healthcare are central goals of the Sustainable Development Goals (SDGs). Despite global progress, Nigeria continues to report one of the highest under-five mortality rates and the lowest access to maternal healthcare worldwide.

**Objectives:**

This study examines whether maternal education is associated with under-five mortality and access to maternal care in Nigeria. It also investigates whether these associations differ between rural and urban areas and whether they are explained by socioeconomic, ethnic, and religious factors. In addition, we assess whether literacy exerts an independent influence alongside formal education.

**Methodology:**

Using the 2021 Nigeria Malaria Indicator Survey (MIS) and a sample of 10,820 women of reproductive age, we employ a bivariate probit framework supplemented by propensity score matching (PSM) to examine the robustness of the findings.

**Results:**

Higher levels of maternal education, particularly secondary schooling, were significantly associated with a 2.08 ppt reduction in under-five mortality and a 6.74 ppt increase in access to maternal care. Economic status modestly attenuated the size of the education association with under-five mortality but strengthened it for maternal care, while ethnicity and religion partly explained the observed associations. Literacy displayed similar patterns to formal education when examined separately but did not retain an independent association with either outcome in fully adjusted models or when included alongside education. These findings are robust across alternative specifications.

**Recommendation:**

Economically and culturally tailored policies that improve female educational attainment remain critical for enhancing access to maternal healthcare and reducing child mortality in Nigeria.

## 1. Introduction

Reducing child mortality and ensuring universal access to maternal healthcare remain critical global health priorities under the United Nations Sustainable Development Goals (SDGs). Specifically, SDG 3 targets a reduction in the under-five mortality rate to 25 deaths per 1,000 live births in every country by 2030 [1]. Although global under-five mortality has declined significantly from 93 per 1,000 in 1990–37 in 2022 [2], progress in Nigeria has been markedly slower. In 2023, the country recorded 111 under-five deaths per 1,000 live births [3], placing it among the top five countries with the highest child mortality and accounting for 15% of global under-five deaths, despite making up just 2.78% of the world’s population.

Neonatal disorders, malaria, diarrhoea, and lower respiratory infections remain the leading causes of under-five mortality in Nigeria, jointly accounting for over 73% of such deaths (Statista, 2024). One factor that has consistently emerged in global health literature as an important determinant of improved child health outcomes is education. Higher educational attainment improves individuals’ understanding of health-promoting behaviours and facilitates better use of healthcare services [4,5]. Numerous studies have shown strong associations between educational attainment and health outcomes [6,7,8], with maternal education particularly emphasized as a key predictor of child survival [9,10,11,12].

Maternal education has been shown to be associated with child health through several potential pathways, including health knowledge, economic empowerment, and health service utilization. Among these, access to maternal care such as antenatal care, skilled birth attendance, and postnatal services, is a critical mechanism [13,14]. Yet, despite the potential importance of this pathway, access to maternal care has received limited attention in studies linking maternal education to under-five mortality in Nigeria.

Disparities in accessing maternal healthcare persist in Nigeria, particularly along urban-rural, socioeconomic, and educational dimensions [15]. Geographic barriers, financial constraints, and cultural beliefs continue to limit healthcare access. Despite the introduction of the National Health Insurance Scheme in 2005 and its replacement with the National Health Insurance Authority (NHIA) Act in 2022, only about 10% of the population is currently covered by the NHIA. Out-of-pocket payments dominate healthcare financing, representing 76.24% of health expenditures in 2021 (Statista, 2024).

Further complicating the issue is Nigeria’s low level of female education. Over 50% of girls are out of school at the basic education level, and 7.6 million girls are currently not enrolled in school [16]. This widespread educational deprivation limits women’s ability to access and effectively use maternal healthcare services, contributing to poor maternal and child health outcomes. Moreover, Nigeria’s diverse ethnic and religious landscape may shape the effectiveness of maternal education in reducing child mortality, suggesting the need for context-specific analyses [17,18].

Existing studies have separately estimated the association of maternal education with access to maternal care and child survival in low- and middle-income settings [e.g., 19,20,21,22,23,24,25,26,27]. In Nigeria where healthcare financing is dominated by out-of-pocket payments and insurance coverage is limited, maternal education may influence child survival not only through knowledge, autonomy, and behavioural mechanisms but also through women’s capacity to navigate financial barriers to care. Existing studies rarely embed maternal education within the broader health-financing constraints that shape households’ ability to afford antenatal, delivery, and postnatal services. By analysing maternal education and maternal healthcare access within a context characterised by high user fees and limited financial risk protection, this study offers evidence on how education intersects with economic barriers to shape child survival in low-resource settings. In addition, the separate, single-equation models largely used by previous studies do not account for the fact that access to maternal care and under-five mortality are jointly shaped by shared socioeconomic, cultural, and behavioural factors. Separate models therefore risk misstating the independent association of maternal education with access to maternal care and child mortality by not accounting for correlated unobserved heterogeneity that simultaneously shape both outcomes. This study addresses this gap by using a bivariate probit model that explicitly accounts for correlated unobserved heterogeneity, enabling a clearer separation of pathways and providing more evidence on how maternal education is associated with maternal care uptake and under-five mortality.

Guided by prior empirical evidence, we hypothesise that maternal education is associated with both improved access to maternal care and lower under-five mortality. We posit that these associations operate through several mechanisms. First, educated mothers may possess greater health knowledge and awareness of pregnancy-related risks, are better able to interpret symptoms and care recommendations, and more effectively navigate the health system. Education may also enhance women’s autonomy, decision-making power, and economic opportunities, enabling them to overcome financial and logistical barriers to maternal care. These mechanisms are particularly salient in Nigeria’s healthcare financing context, where high out-of-pocket payments can hinder access to skilled maternal care, especially for women with limited education and limited economic resources. Second, the strength of these associations may be shaped by socioeconomic and sociocultural factors such as wealth, ethnicity, religion, and rural–urban divide. These hypotheses provide a conceptual structure for interpreting the empirical results within Nigeria’s broader healthcare, financing, and sociocultural environment.

### 1.1. Institutional background

Nigeria’s healthcare system features both public and private providers, with significant regional disparities in access and quality. Organized by a three-tier government structure – local, state, and federal, the system aims to offer comprehensive care nationwide. Primary healthcare forms the backbone of the health system, focusing on accessible, affordable services, such as antenatal, skilled birth attendance, and postnatal care, mainly delivered through Primary Health Centres, Health Posts, and Community Health Workers. However, primary care often struggles with inadequate funding, insufficient staff, and poor infrastructure, especially in rural areas, pushing some patients to travel for essential services.

Secondary healthcare provides more specialized care in urban-based General Hospitals and Specialist Clinics, covering areas like obstetrics, paediatrics, and surgery. Due to limited secondary services in rural regions, patients, particularly pregnant women, face higher risks when unable to access advanced care locally. Tertiary healthcare, the system’s most advanced tier, includes Teaching Hospitals, Federal Medical Centres, and Specialized Hospitals focused on specific medical fields, such as cardiology and orthopaedics, providing complex treatments and training for medical professionals.

Healthcare administration is divided among government bodies, including the Federal Ministry of Health, State Health Ministries, the National Health Insurance Authority (NHIA) formerly known as the National Health Insurance Scheme (NHIS) for universal health coverage, and the National Primary Health Care Development Agency, which supports immunization and primary care initiatives. Despite efforts, systemic challenges in coordination, funding, and access continue to affect equitable healthcare delivery across Nigeria.

Healthcare financing in Nigeria is central to improving health outcomes and moving toward universal health coverage. Funding comes from various sources, including government budgets, the NHIA, out-of-pocket payments, donor funds, and private sector contributions. The NHIS, launched in 2005 and replaced by the NHIA in 2022, relies on a contributory model where employers and employees share costs. Despite the replacement of the NHIS with the NHIA, barely 10% of the population is covered.

Out-of-pocket payments are the primary funding source for healthcare, comprising over 76.24% of total health expenditure in 2021 (Statista, 2024). This heavy reliance on individual payments poses challenges for low-income individuals and contributes to unequal healthcare access. Donor aid, covering about 7.86% of healthcare costs, plays a significant role, particularly for disease-specific initiatives. Private health insurance and private sector support also contribute but cover a small percentage of the population, with less than 5% estimated to hold private insurance.

Sustainable healthcare financing remains a challenge, requiring innovative solutions to bridge funding gaps and ensure equitable access to essential services. Expanding the NHIA coverage to the informal sector and developing alternative financing models are recommended strategies to help Nigeria achieve universal health coverage and improve healthcare access, particularly for vulnerable populations like children and pregnant women.

## 2. Materials and methods

### 2.1. Data and variables

This study draws on data from the 2021 Nigeria Malaria Indicator Survey (MIS), conducted as part of the Demographic and Health Survey (DHS) program. The DHS provides nationally representative data for monitoring health, population, and nutrition indicators across Nigeria’s 36 states and the Federal Capital Territory. The 2021 MIS employed a two-stage stratified random sampling technique to ensure national and subnational representativeness.

The survey collected comprehensive information on household characteristics, maternal background, and children’s birth histories and survival status. The dataset includes 70,428 individuals from 13,727 households, with detailed responses from 14,476 women aged 15–49. Of these, 10,988 women reported having given birth and were eligible for this study. Among them, 70.3% reside in rural areas and 29.7% in urban areas. We excluded 168 observations with missing values for the age of the household head using listwise deletion, giving a final analytical sample of 10,820 women. No other variables used in the analysis had missing observations. The choice of listwise deletion reflects the relatively small proportion of missing observations (approximately 1.5% of eligible respondents) and the absence of systematic patterns of missingness across key covariates. We acknowledge that if the excluded cases differ systematically from the retained sample (in socioeconomic status or demographic characteristics), it may introduce a modest degree of selection bias. Nonetheless, given the limited scale and random distribution of the missingness, the implications for sample representativeness and inference are expected to be minimal.

#### 2.1.1. *Dependent variables.*

Under-five mortality is measured as a binary variable equal to 1 if the mother has experienced the death of at least one child under age five, and 0 otherwise. This is based on the child’s survival status reported in the birth history section. Access to maternal care is also a binary variable coded 1 if the respondent received both antenatal and postnatal care for her most recent birth, and 0 if either service was not accessed.

#### 2.1.2. *Maternal education.*

Categorized into four levels: no education, primary, secondary, and tertiary. These categories capture the highest level of formal schooling completed by the respondent. “No education” serves as the reference group in regression analyses. Differences in health outcomes are expected to vary by level of education.

#### 2.1.3. *Literacy.*

Included as a binary variable coded 1 if the mother reports being able to read, and 0 otherwise. Although literacy may strongly correlate with formal educational attainment, it captures a distinct dimension of functional skills that may influence health-related decision-making independently of years of schooling [28]. We first treat literacy separately as a distinct alternative to formal education before adding it as a covariate to assess whether the ability to read mediates or confounds the association between education and the two outcomes of interest. This approach allows us to distinguish between the effect of educational credentials and the effect of functional reading ability, while recognising that literacy may partially lie on the causal pathway from schooling to health behaviours. We therefore interpret the literacy-adjusted model as exploratory rather than causal, aimed at understanding whether literacy provides explanatory value beyond formal education.

#### 2.1.4. *Maternal age.*

Grouped into five categories: 15–24, 25–29, 30–34, 35–39, and 40–49 years. This allows the analysis to account for potential non-linear relationships between age and child survival or maternal care utilization.

#### 2.1.5. *Location of residence.*

Classified as urban or rural, based on DHS definitions. These reflect differences in infrastructure, economic activity, and access to services. A binary variable is used, coded 1 for urban areas and 0 for rural areas.

#### 2.1.6. *Wealth index.*

Derived from household asset ownership, housing quality, and access to water and sanitation. Households are ranked into three quintiles: poor, middle, and rich. Wealthier households are generally expected to have lower under-five mortality and greater healthcare utilization.

#### 2.1.7. *Family size.*

Refers to the number of people living in the household under the same domestic arrangement, including both relatives and non-relatives, and recognizing a common household head.

#### 2.1.8. *Age of household head.*

Captures the current age of the person designated as head of the household. This variable is included to explore whether generational differences among household heads influence maternal or child health outcomes.

#### 2.1.9. *Ethnicity.*

Recoded into six categories: Fulani, Hausa, Igbo, Yoruba, minority ethnic groups, and others. This reflects the ethnolinguistic diversity of Nigeria and allows examination of sociocultural variation in health behaviours.

#### 2.1.10. *Religion.*

Categorized as Christian, Muslim, or Traditionalist. Since religious beliefs can associate with attitudes toward education and modern healthcare, this variable helps contextualize the associations observed.

The description of variables, measurements and source of data used in this study are summarised in Table 1.

**Table 1:** Description and measurements of variables.

Variable Name	Label	Coding Type
Under-five deaths	At least a child died before 5^th^ birthday	Dummy (1 = yes, 0 = no)
Maternal education	The highest level of educational attainment	Ordinal (0 = no education, 1 = primary, 2 = secondary, 3 = tertiary)
Literacy	Able to read	Dummy (1 = yes and 0 = no)
Mother’s age	Mother’s 5-year age groups	Ordinal (1 = 15–24, 2 = 25–29, 3 = 30–34, 4 = 35–39, 5 = 40–49)
Access to maternal care	Accessed prenatal and postnatal care	Dummy (1 = yes, 0 = no)
Location of residence	Type of location of residence	Dummy (1 = urban, 0 = rural)
Wealth index	Household economic status	Ordinal (1 = poor, 2 = middle, 3 = rich)
Family size	Number of household members	Discrete (2–46)
Age of household head	Age of household head	Discrete (17–95)
Ethnicity	Ethnicity	Nominal (1 = Others, 2 = Minorities, 3 = Fulani, 4 = Yoruba, 5 = Igbo, 6 = Hausa)
Religion	Religion	Nominal (1 = Traditionalist, 2 = Christian, 3 = Muslim)

DHS. https://dhsprogram.com/data/dataset/Nigeria_MIS_2021.cfm?flag=0

### 2.2. *Empirical specification*

The Grossman’s model, which emphasizes education as a determinant of health production, provides a robust theoretical framework to understand how maternal education is associated with access to maternal care and under-five mortality. The model conceptualizes health as both consumption and investment goods, where individuals produce their health, combining various inputs such as medical care and education to maintain or improve their health status [4,29]. This model particularly views education as pivotal in influencing health production efficiency as better-educated individuals are presumed to make more informed health-related decisions, allocate resources effectively, and adopt healthier behaviours [30,31], Hoffmann and Lutz, 2018, Begerow and Jürges, 2021]. Relying on this model, we posit that educated mothers are more likely to possess and apply knowledge about nutrition, hygiene, maternal and preventive cares, leading to better health outcomes for their children.

To investigate the association between education and both under-five mortality and access to maternal care, we use a bivariate probit model [32,33,34,35]:


$$y_{\text{1}}=\alpha +x_{i}^{\prime }\beta +z_{i}^{\prime }\delta +r_{i}^{\prime }\theta +\varepsilon_{\text{1}}$$ (1)

$$y_{\text{2}}=\alpha +x_{i}^{\prime }\beta +z_{i}^{\prime }\delta +r_{i}^{\prime }\theta +\varepsilon_{\text{2}}$$ (2)

where $y_{\text{1}}$ is the probability of under-five mortality (with $y_{\text{1}}$ = 1 if a child died before the age of five and $y_{\text{1}}\;$= 0 if otherwise) and $y_{\text{2}}$ is the probability of accessing maternal healthcare (with $y_{\text{2}}$ = 1 if a mother is enrolled for antenatal and postnatal cares and $y_{\text{2}}\;$= 0, if otherwise); $x_{i}\;\text{is}$ a vector of variables related to maternal education; $z_{i}$ is a vector of explanatory variables (wealth index, mother’s age, family size, age of household head and location of residence); $r_{i}\;\text{is}\;\text{a}\;$vector of contextual variables (religion and ethnicity); $\varepsilon_{\text{1}}$ and $\varepsilon_{\text{2}}$ are unobserved factors assumed to follow a joint normal distribution with zero mean, constant variance, and a correlation coefficient of ρ. We estimate the coefficients by maximising the log-likelihood function and then compute the marginal effects of all regressors [36,33,Torres et al., 2016,^35^].

The choice of a bivariate probit model is because there may be unobserved household characteristics that are simultaneously associated with both under-five mortality and maternal healthcare use. The bivariate probit model allows the error terms of the two models to be correlated, enabling a formal test of whether unobserved characteristics are simultaneously associated with both outcomes. The statistical significance of the estimated correlation coefficient (ρ) across all specifications confirms that modelling the two outcomes jointly provides a better representation of the data than estimating them separately. We also compared the predicted probabilities of probit and logit models for both outcomes and find that correlation between logit-based and probit-based predicted probabilities is 0.999. In addition, we compared marginal effects for logit-based and probit-based regressions, and the results are similar in both magnitude and direction (see S1 Appendix). Therefore, our findings are robust to the choice between logit and probit models.

We run four specifications by gradually adding different sets of control variables. In the first specification, we only control for age. In the second specification, we also control for wealth. In the third specification we include all the control variables in vector $z_{i}$. In the fourth specification, we control for religion and ethnicity in vector $r_{i}$.

## 3. Results

### 3.1. Summary statistics

Table 2 presents summary statistics. For the overall sample, under-five mortality rate is 3.1%. About 79.5% of women accessed maternal care. 42.7% of mothers have no education, 14.9% have primary education, 31.7% have secondary education, and 10.6% have higher education. 49.4% of women are considered literate and can read. 23.3% of women are 15–24 years old, 27.3% are 25–29 years old, 23.7% are 30–34 years old, 16.1% are 35–39 and 9.6% are 40–49 years old. Regarding economic status, 40.5% of women are poor, 40.5% have middle wealth, and 19% are rich. The sample is diverse in terms of ethnicity and religion. 26.5% are from Hausa ethnic group, 12.3% are from Igbo ethnic group, 9.3% are Fulani, 8.7% are Yoruba, 26.5% are from other minority ethnic groups, and 16.5% are from other ethnic groups. 56.8% are from Muslim communities, 42.6% from Christian communities, and 0.6% are Traditionalists.

**Table 2:** Summary statistics.

Variables	Mean (Overall)	Mean (Rural)	Mean (Urban)	Min.	Max.
Under-five mortality rate	0.031	0.035	0.021	0	1
Access to maternal care	0.205	0.197	0.226	0	1
Literacy (=1 able to read)	0.494	0.411	0.693	0	1
Location (=1 if urban)	0.297	--	--	0	1
Family size	7.43	7.72	6.7	1	46
Age of household head	42	42	42	17	95
**Maternal Education** No education Primary education Secondary education Tertiary education	0.427 0.149 0.317 0.106	0.511 0.15 0.281 0.058	0.230 0.147 0.402 0.221	0 0 0 0	1 1 1 1
**Maternal age (years)** 15-24 25-29 30-34 35-39 40-49	0.233 0.273 0.237 0.161 0.096	0.263 0.275 0.226 0.147 0.090	0.162 0.269 0.264 0.195 0.110	0 0 0 0 0	1 1 1 1 1 1
**Wealth index** Poor Middle Rich	0.405 0.405 0.190	0.510 0.390 0.100	0.157 0.442 0.402	0 0 0	1 1 1
**Ethnicity** Fulani Igbo Hausa Yoruba Minorities Others	0.093 0.123 0.265 0.087 0.268 0.165	0.110 0.123 0.285 0.048 0.267 0.167	0.052 0.122 0.218 0.179 0.271 0.159	0 0 0 0 0 0	1 1 1 1 1 1
**Religion** Christian Islamic Traditional	0.426 0.568 0.006	0.411 0.583 0.006	0.462 0.534 0.004	0 0 0	1 1 1
**Obs.**	**10,820**	**7,611**	**3,209**		

Demographic and Health Survey. https://dhsprogram.com/data/dataset/Nigeria_MIS_2021.cfm?flag=0

There are marked differences between urban and rural areas. Under-five mortality is higher in rural areas (3.5% vs 2.1%) and maternal care access is lower (19.7% vs 22.6%). Education is lower (23.0% vs 51.1% of women with no education), and maternal age is lower (26.3% vs 16.2% of women are in the 15–24 age group). Wealth is much lower in rural areas with 51% of women being poor against 15.7% in urban areas.

**Fig 1:**
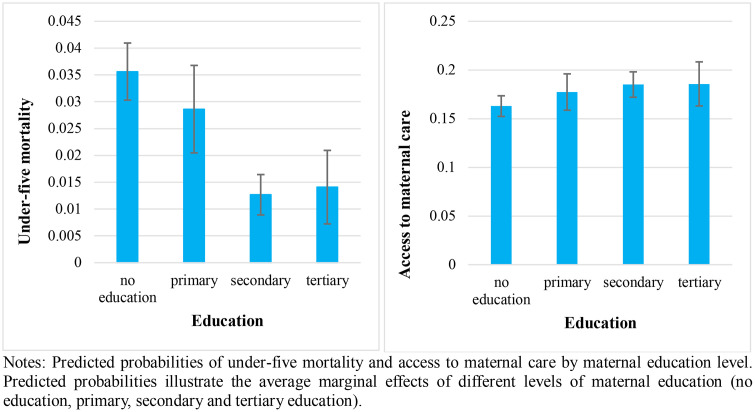
Under-five mortality rate and maternal health care use by maternal education level, with 95% confidence intervals.

To visually summarise how maternal education relates to both outcomes, Fig 1 presents the predicted probabilities of under-five mortality and access to maternal care by education level with the 95% confidence interval bars. Compared to mothers with no education, under-five mortality declines markedly from mothers with primary to those with secondary or tertiary schooling, whereas access to maternal care increases steadily from primary to higher education levels.

### 3.2. Regression results: maternal education, access to maternal care and under-five mortality

Tables 3 and 4 report the results of bivariate probit regressions on how education is associated with under-five mortality and access to maternal care respectively. The models incrementally add different covariates to see what factors explain the association between education and under-five mortality or maternal care. Marginal effects are provided. We assessed model adequacy using the McFadden pseudo-R² and the Wald test of the correlation coefficient (ρ) between the two equations. The pseudo-R² for the full bivariate probit specification is 0.053, indicating a modest but meaningful improvement in fit relative to the null model, which is typical for nonlinear models with binary outcomes in survey data. The Wald test of rho is statistically significant (*χ²* = 12.24, *p* = 0.0005), rejecting the null hypothesis ρ = 0 and confirming that the two outcomes share correlated unobserved factors. This validates our choice of a bivariate probit model rather than separate univariate probits.

**Table 3:** Regression results: maternal education and under-five mortality.

Model	(1)	(2)	(3)	(4)
**Education (‘No education’ as reference)**				
Primary education	−0.0080	−0.0060	−0.0061	0.0021
	(0.005)	(0.005)	(0.005)	(0.005)
Secondary education	−0.0208***	−0.0151***	−0.0156***	−0.0013
	(0.004)	(0.004)	(0.005)	(0.005)
Tertiary education	−0.0190***	0.0054	0.0056	0.0116
**Age (‘15-24’ as reference)**	(0.005)	(0.008)	(0.008)	(0.010)
25-29	−0.0143***	−0.0133***	−0.0140***	−0.0092*
	(0.005)	(0.005)	(0.005)	(0.005)
30-34	−0.0155***	−0.0144***	−0.0154***	−0.0099*
	(0.005)	(0.005)	(0.005)	(0.005)
35-39	−0.0234***	−0.0219***	−0.0233***	−0.0161***
	(0.005)	(0.005)	(0.005)	(0.006)
40-49	−0.0086	−0.0070	−0.0098	−0.0036
**Wealth index (‘Poor’ as reference)**	(0.007)	(0.007)	(0.007)	(0.007)
Middle		−0.0021	−0.0013	0.0030
		(0.004)	(0.004)	(0.004)
Rich		−0.0177***	−0.0158***	0.0099*
		(0.005)	(0.005)	(0.006)
**Family size**			−0.0005	−0.0012**
			(0.0005)	(0.0005)
**Location (urban = 1 & rural = 0)**			−0.0057	−0.0100**
			(0.004)	(0.004)
**Age of household head**			0.0003	0.0003*
**Religion (‘Traditionalist’ as reference)**			(0.0002)	(0.0002)
Christian				−0.0156
				(0.027)
Muslim				−0.0083
**Ethnicity (‘Others’ as reference)**				(0.027)
Minorities				0.0026
				(0.005)
Fulani				0.0286***
				(0.009)
Yoruba				0.0236***
				(0.007)
Igbo				−0.0158***
				(0.004)
Hausa				−0.0012
				(0.006)
**Obs.**	**10820**	**10820**	**10820**	**10820**

These results are based on average marginal effect from the bivariate probit regression for maternal education and under-5 mortality. Robust standard errors in parentheses. rho = −0.14 is residual correlation coefficient between under-5 mortality and access to maternal care. Wald test of rho = 0: chi2(1) = 12.2364; p-values of rho’s Wald test = 0.0005. Pseudo-R² of 0.053. *** p < 0.01, ** p < 0.05, * p < 0.1

**Table 4:** Regression results: maternal education and access to maternal care.

Model	(1)	(2)	(3)	(4)
**Education (‘No education’ as reference)**				
Primary education	0.0422***	0.0538***	0.0516***	0.0286**
	(0.011)	(0.012)	(0.012)	(0.012)
Secondary education	0.0674***	0.0949***	0.0895***	0.0516***
	(0.009)	(0.011)	(0.012)	(0.012)
Tertiary education	0.0343**	0.0774***	0.0700***	0.0289
**Age (‘15-24’ as reference)**	(0.0013)	(0.017)	(0.017)	(0.017)
25-29	0.0325***	0.0349***	0.0344***	0.0299***
	(0.010)	(0.009)	(0.010)	(0.010)
30-34	0.0595***	0.0627***	0.0609***	0.0556***
	(0.010)	(0.010)	(0.011)	(0.011)
35-39	0.1466***	0.1524***	0.1470***	0.1363***
	(0.013)	(0.013)	(0.013)	(0.013)
40-49	0.2296***	0.2349***	0.2232***	0.2079***
**Wealth index (‘Poor’ as reference)**	(0.017)	(0.016)	(0.018)	(0.018)
Middle		−0.0323***	−0.0356***	−0.0376***
		(0.010)	(0.011)	(0.010)
Rich		−0.0596***	−0.0661***	−0.0583***
		(0.014)	(0.014)	(0.014)
**Family size**			−0.0029***	−0.0010
			(0.001)	(0.001)
**Location (urban = 1 & rural = 0)**			0.0115	0.0195**
			(0.009)	(0.009)
**Age of household head**			0.0010**	0.0012***
**Religion (‘Traditionalist’ as reference)**			(0.0004)	(0.0004)
Christian				0.0600
				(0.051)
Muslim				−0.0458
**Ethnicity (‘Others’ as reference)**				(0.050)
Minorities				0.0461***
				(0.012)
Fulani				−0.0260
				(0.017)
Yoruba				0.0145
				(0.014)
Igbo				−0.0816***
				(0.012)
Hausa				−0.0131
				(0.016)
**Obs.**	**10820**	**10820**	**10820**	**10820**

These results are based on average marginal effect from the bivariate probit regression for maternal education and access to maternal care. Robust standard errors in parentheses. rho = −0.14 is residual correlation coefficient between under-5 mortality and access to maternal care. Wald test of rho = 0: chi2(1) = 12.2364; p-values of rho’s Wald test = 0.0005. Pseudo-R² of 0.053. *** p < 0.01, ** p < 0.05, * p < 0.1

In the first regressions in Column (1) of Tables 3 and 4, we only control for age. We find that secondary and tertiary education are significantly associated with lower under-five mortality relative to no education (Table 3), whereas primary, secondary and tertiary education are significantly associated with improved maternal healthcare use (Table 4). In more detail, secondary education is associated with a 2.08 percentage point (ppt) reduction in the probability of under-five mortality, and tertiary education with a 1.9 ppt reduction, relative to women with no education. Primary, secondary and tertiary education is significantly associated with a higher probability of access to maternal care by 4.22 ppt, 6.74 ppt, and 3.43ppt, respectively, compared to mothers with no education.

Maternal age is generally negatively associated with under-five mortality. Women aged 25–29, 30–34, and 35–39 have 1.43 ppt, 1.55 ppt, and 2.34 ppt, respectively, lower under-five mortality, compared to the youngest group (15–24). Maternal healthcare use is instead positively associated with age. Women aged 25–29, 30–34, 35–39, and 40–49 have 3.25 ppt, 5.95 ppt, 14.66 ppt, and 22.96 ppt, respectively, higher probability of accessing maternal care than women aged 15–24.

In the second regressions in Column (2) of Tables 3 and 4 we control for economic status by adding the wealth index. Given that wealth is positively associated with education, we find that the education gradient dropped after controlling for wealth. Secondary education remains significantly associated with under-five mortality (Table 3), but its marginal effect drops by 1.51 ppt (relative to 2.08 ppt in Column (1)). As expected, wealth is negatively associated with under-five mortality – households in the rich class are significantly negatively associated with under-five mortality by 1.7 ppt.

Adding wealth in the regression on maternal care increases the association between education and maternal care (Column (2) of Table 4). Primary, secondary and tertiary education is positively associated with the probability of maternal care by 5.38 ppt, 9.49 ppt and 7.74 ppt (relative to 4.22 ppt, 6.74 ppt and 3.43 ppt in Column (1)). Instead, wealth is negatively associated with access to maternal care, with marginal effects of 3.23 ppt and 5.96 ppt for middle and rich households, respectively. This suggests that it is education rather than wealth that associates with higher maternal healthcare use.

In Column (3) of Tables 3 and 4, we add other household variables such as family size, location of residence (urban vs rural), and age of household head. However, the inclusion of these variable does not significantly alter the findings in Column (2). None of the other household variables is significantly associated with under-five mortality. For maternal healthcare use, only family size and age of household heads are statistically significant. One additional household member is negatively associated with maternal care by 0.29 ppt, and one extra year in the age of household head is positively associated with maternal care by 0.1 ppt.

Last, in Column (4) of Tables 3 and 4, we include religion and ethnicity and show that they are important factors in explaining the education gradient in under-five mortality, and partially the education gradient in maternal healthcare use. After inclusion of religion and ethnicity, education is not significantly associated with under-five mortality. The probability of under-five mortality is significantly higher by 2.86 ppt for Fulani households, and 2.36 ppt for Yoruba households, but significantly lower by 1.58 ppt for Igbo households, compared to other ethnic nationalities. The lack of association between education and mortality can be explained by the correlation between education and wealth with religion and ethnicity, as also confirmed by the lower coefficient of wealth.

The magnitude of the association between education and maternal care significantly shrinks on the introduction of religion and ethnicity (Table 4). Primary, secondary and tertiary education coefficients drop to 2.86 ppt, 5.16 ppt and 2.89 ppt, respectively, as against the previous coefficients (5.16 ppt, 8.85 ppt and 7 ppt). However, only minority and Igbo ethnic groups are significantly associated with better use of maternal healthcare. The minority group is positively associated with maternal care use by 4.61 ppt, while Igbo ethnic group is negatively associated with maternal care use by 8.16 ppt, compared to other ethnic groups.

We have used bivariate probit regressions to investigate the association between education and under-five mortality or maternal care access. Across the four specifications in Columns (1) to (4), the correlation coefficients of the error terms (rho) are stable and equal to −0.14, −0.15, −0.15, and −0.14. The Wald test suggests that these are statistically significant. This negative correlation suggests that there are unobserved factors that are simultaneously positively associated with maternal care and negatively associated with under-five mortality. In other words, women who are (unobservably) more likely to use maternal healthcare also have a reduced probability of experiencing under-five mortality of their children.

### 3.3 Comparison between urban and rural areas

Tables 5 and 6 provide results for under-five mortality when the sample is split between rural and urban areas, respectively. The results for the rural sample are very similar to those presented in Tables 3. Age groups, wealth, family size and ethnicities are associated with under-five mortality, with almost the same coefficients as in the overall sample regressions.

**Table 5:** Regression results for rural areas: under-five mortality.

Model	(1)	(2)	(3)	(4)
**Education (‘No education’ as reference)**				
Primary	−0.0089	−0.0083	−0.0086	0.0023
	(0.006)	(0.006)	(0.006)	(0.006)
Secondary	−0.0258***	−0.0221***	−0.0226***	−0.0047
	(0.004)	(0.005)	(0.005)	(0.006)
Tertiary	−0.0216***	−0.0063	−0.0068	0.0187
**Age (‘15-24’ as reference)**	(0.008)	(0.013)	(0.014)	(0.019)
25-29	−0.0131**	−0.0122**	−0.0134**	−0.0079
	(0.006)	(0.006)	(0.006)	(0.006)
30-34	−0.0150**	−0.0138**	−0.0157**	−0.0101
	(0.006)	(0.006)	(0.007)	(0.006)
35-39	−0.0288***	−0.0276***	−0.0299***	−0.0228***
	(0.006)	(0.006)	(0.007)	(0.007)
40-49	−0.0105	−0.0091	−0.0132	−0.0079
**Wealth index (‘Poor’ as reference)**	(0.008)	(0.008)	(0.008)	(0.009)
Middle		0.0009	0.0006	0.0061
		(0.005)	(0.005)	(0.005)
Rich		−0.0249***	−0.0249***	−0.0199**
		(0.007)	(0.007)	(0.008)
**Family size**			−0.0004	−0.0011*
			(0.001)	(0.0006)
**Age of household head**			0.0002	0.0004*
**Religion (‘Traditionalist’ as reference)**			(0.0001)	(0.0002)
Christian				−0.0003
				(0.023)
Muslim				0.0156
**Ethnicity (‘Others’ as reference)**				(0.023)
Minorities				0.0050
				(0.006)
Fulani				0.0258***
				(0.010)
Yoruba				0.0205***
				(0.007)
Igbo				−0.0177***
				(0.006)
Hausa				−0.00004
				(0.010)
**Obs.**	**7611**	**7611**	**7611**	**7611**

These results are based on average marginal effect from the bivariate probit regression for under-5 mortality in rural areas. Robust standard errors in parentheses. rho = −0.12: residual correlation coefficient between under-5 mortality and access to maternal care. P-value for the rho’s Wald test is 0.010. *** p < 0.01, ** p < 0.05, * p < 0.1

**Table 6:** Regression results for urban areas: under-five mortality.

Model	(1)	(2)	(3)	(4)
**Education (‘No education’ as reference)**				
Primary	0.0039	0.0043	0.0039	0.0056
	(0.009)	(0.009)	(0.009)	(0.008)
Secondary	−0.0001	0.0040	0.0026	0.0080
	(0.007)	(0.007)	(0.008)	(0.007)
Tertiary	−0.0015	0.0066	0.0042	0.0099
**Age (‘15-24’ as reference)**	(0.008)	(0.009)	(0.010)	(0.009)
25-29	−0.0138	−0.0127	−0.0119	−0.0081
	(0.009)	(0.008)	(0.008)	(0.007)
30-34	−0.0121	−0.0110	−0.0103	−0.0046
	(0.009)	(0.009)	(0.008)	(0.008)
35-39	−0.0093	−0.0077	−0.0075	−0.0007
	(0.009)	(0.009)	(0.009)	(0.009)
40-49	0.0007	0.0024	0.0014	0.0100
**Wealth index (‘Poor’ as reference)**	(0.012)	(0.012)	(0.012)	(0.013)
Middle		−0.0004	0.0010	−0.0021
		(0.010)	(0.009)	(0.009)
Rich		−0.0104	−0.0098	−0.0091
		(0.010)	(0.010)	(0.010)
**Family size**			−0.0014	−0.0019*
			(0.001)	(0.001)
**Age of household head**			0.0003	0.0002
**Religion (‘Traditionalist’ as reference)**			(0.0003)	(0.0003)
Christian				−0.1035
				(0.103)
Muslim				−0.1122
**Ethnicity (‘Others’ as reference)**				(0.103)
Minorities				−0.0014
				(0.007)
Fulani				0.0394*
				(0.023)
Yoruba				0.0352**
				(0.015)
Igbo				−0.0109*
				(0.006)
Hausa				−0.0031
				(0.007)
**Obs.**	**3209**	**3209**	**3209**	**3209**

These results are based on average marginal effect from the bivariate probit regression for under-5 mortality in urban areas. Robust standard errors in parentheses. rho = −0.24: residual correlation coefficient between under-5 mortality and access to maternal care. P-value for the rho’s Wald test is 0.002.*** p < 0.01, ** p < 0.05, * p < 0.1

Secondary education is significantly and negatively associated with under-five mortality in Columns (1)-(3) of Table 5. Adding economic status in Column (2) reduces the marginal effect of secondary education from 2.58 ppt to 2.21 ppt. One additional household member significantly associates with lower under-five mortality by 0.19 ppt among urban households compared to rural households (0.11 ppt). We find no association between education and mortality in the urban sample (Table 6).

Tables 7 and 8 provide results for maternal care when the sample is again split between rural and urban areas, respectively. Primary education is significant in rural sample regressions (Table 7) but not in urban sample regressions (Table 8). Secondary and tertiary education are both significant for rural and urban sample regressions. When controlling for age in Column (1), women with secondary education in urban and rural areas are 8.84 ppt and 6.01 ppt more likely to access maternal healthcare relative to no education. However, tertiary education is only significant for women in urban areas with 5.72 ppt positively associated with access to maternal care.

**Table 7:** Regression results for rural areas: access to maternal care.

Model	(1)	(2)	(3)	(4)
**Education (‘No education’ as reference)**				
Primary	0.0507***	0.0627***	0.0611***	0.0349**
	(0.014)	(0.014)	(0.014)	(0.014)
Secondary	0.0601***	0.0938***	0.0891***	0.0525***
	(0.011)	(0.014)	(0.014)	(0.015)
Tertiary	0.0113	0.0763***	0.0713***	0.0253
**Age (‘15-24’ as reference)**	(0.019)	(0.025)	(0.025)	(0.024)
25-29	0.0447***	0.0478***	0.0470***	0.0421***
	(0.011)	(0.011)	(0.011)	(0.011)
30-34	0.0576***	0.0619***	0.0599***	0.0549***
	(0.012)	(0.012)	(0.012)	(0.012)
35-39	0.1272***	0.1345***	0.1287***	0.1191***
	(0.015)	(0.015)	(0.016)	(0.016)
40-49	0.2091***	0.2151***	0.2037***	0.1929***
**Wealth index (‘Poor’ as reference)**	(0.020)	(0.020)	(0.021)	(0.021)
Middle		−0.0362***	−0.0374***	−0.0388***
		(0.012)	(0.012)	(0.011)
Rich		−0.0907***	−0.0921***	−0.0746***
		(0.016)	(0.016)	(0.017)
**Family size**			−0.0025**	−0.0006
			(0.001)	(0.001)
**Age of household head**			0.0009**	0.0012***
**Religion (‘Traditionalist’ as reference)**			(0.0004)	(0.0004)
Christian				0.0870*
				(0.052)
Muslim				−0.0155
**Ethnicity (‘Others’ as reference)**				(0.051)
Minorities				0.0400**
				(0.014)
Fulani				−0.0306
				(0.020)
Yoruba				−0.0025
				(0.017)
Igbo				−0.1135***
				(0.014)
Hausa				−0.0118
				(0.023)
**Obs.**	**7611**	**7611**	**7611**	**7611**

These results are based on average marginal effect from the bivariate probit regression for access to maternal care in rural areas. Robust standard errors in parentheses. rho = −0.12: residual correlation coefficient between under-5 mortality and access to maternal care. P-value for the rho’s Wald test is 0.010. *** p < 0.01, ** p < 0.05, * p < 0.1

**Table 8:** Regression results for urban areas: access to maternal care.

Model	(1)	(2)	(3)	(4)
**Education (‘No education’ as reference)**				
Primary	0.0254	0.0272	0.0244	0.0150
	(0.022)	(0.023)	(0.023)	(0.024)
Secondary	0.0884***	0.0947***	0.0878***	0.0580**
	(0.018)	(0.022)	(0.022)	(0.024)
Tertiary	0.0572***	0.0678**	0.0584**	0.0278
**Age (‘15-24’ as reference)**	(0.021)	(0.027)	(0.027)	(0.028)
25-29	0.0062	0.0072	0.0086	0.0024
	(0.019)	(0.019)	(0.019)	(0.020)
30-34	0.0681***	0.0693***	0.0699***	0.0625***
	(0.021)	(0.021)	(0.021)	(0.022)
35-39	0.1886***	0.1904***	0.1881***	0.1707***
	(0.024)	(0.024)	(0.025)	(0.026)
40-49	0.2772***	0.2790***	0.2684***	0.2460***
**Wealth index (‘Poor’ as reference)**	(0.030)	(0.030)	(0.033)	(0.033)
Middle		−0.0027	0.0011	0.0039
		(0.025)	(0.025)	(0.025)
Rich		−0.0157	−0.0144	−0.0117
		(0.029)	(0.029)	(0.029)
**Family size**			−0.0050**	−0.0033
			(0.002)	(0.002)
**Age of household head**			0.0014*	0.0016**
**Religion (‘Traditionalist’ as reference)**			(0.0008)	(0.0008)
Christian				−0.0401
				(0.137)
Muslim				−0.1337
**Ethnicity (‘Others’ as reference)**				(0.136)
Minorities				0.0703***
				(0.023)
Fulani				−0.0419
				(0.036)
Yoruba				0.0518*
				(0.028)
Igbo				0.0086
				(0.025)
Hausa				0.0014
				(0.024)
**Obs.**	**3209**	**3209**	**3209**	**3209**

These results are based on average marginal effect from the bivariate probit regression for access to maternal care in urban areas. Robust standard errors in parentheses. rho = −0.24: residual correlation coefficient between under-5 mortality and access to maternal care. P-value for the rho’s Wald test is 0.002. *** p < 0.01, ** p < 0.05, * p < 0.1

Rural women are more likely to access maternal care for both urban and rural areas. Women in urban areas who fall within age groups 30–34, 35–39 and 40–49 are 6.81 ppt, 18.86 ppt and 27.72 ppt more likely to access maternal care while the effect is 5.76 ppt, 12.72 ppt and 20.91 ppt in rural areas, respectively.

Economic status is only statistically significant in rural areas. Women with middle wealth are 3.88 ppt less likely to access maternal care, while those in rich class are 7.46 ppt less likely to access maternal care, relative to those within the poor wealth class. An extra year in the age of household head is significantly positively associated with maternal care by 0.16 ppt in urban areas and by 0.12 ppt in rural areas. Minority ethnic groups living in urban areas are 7.03 ppt more likely to access maternal care than their rural counterparts (4.0 ppt).

Tables 9 and 10 show that the association of literacy (ability to read) with under-five mortality and access to maternal is similar to how formal education associates with both outcomes. However, the magnitude of literacy’s association is somewhat smaller than that of formal education, but the pattern is consistent. With maternal age as the only covariate, being literate is significantly associated with a 1.18 ppt reduction in the probability of under-five mortality and a 3.36 ppt higher likelihood of accessing maternal healthcare than non-literate mothers, compared with non-literate mothers (Column (1) of Tables 9 and 10). On the addition of wealth index (Column 2 of Table 9), the marginal effect drops sharply from 1.18 ppt to 0.32 ppt and becomes non-significant for under-five mortality, indicating that a substantial portion of the apparent protective association between literacy and child survival was explained by wealth differences between literate and non-literate women. However, the marginal effect becomes slightly larger (from 3.36 ppt to 4.31 ppt) and significant for access to maternal care, suggesting that literacy has an independent positive association with access to maternal care (Column (2) of Table 10).

**Table 9:** Regression results: literacy and under-five mortality.

Model	(1)	(2)	(3)	(4)
**Literacy (‘Not able to read’ as reference)**				
Able to read	−0.0118***	−0.0032	−0.0033	0.005
	(0.003)	(0.004)	(0.004)	(0.004)
**Age (‘15-24’ as reference)**				
25-29	−0.0147***	−0.0131***	−0.0138***	−0.0091*
	(0.005)	(0.005)	(0.005)	(0.005)
30-34	−0.0160***	−0.0137***	−0.0148***	−0.0093
	(0.005)	(0.005)	(0.005)	(0.005)
35-39	−0.0241***	−0.0212***	−0.0227***	−0.0153**
	(0.005)	(0.005)	(0.005)	(0.006)
40-49	−0.0079	−0.0055	−0.0083	−0.0027
**Wealth index (‘Poor’ as reference)**	(0.007)	(0.007)	(0.007)	(0.007)
Middle		−0.0077*	−0.0067	0.0017
		(0.004)	(0.004)	(0.004)
Rich		−0.0229***	−0.0209***	−0.0095
		(0.005)	(0.005)	(0.005)
**Family size**			−0.0003	−0.0012**
			(0.0005)	(0.0005)
**Location (urban = 1, rural = 0)**			−0.0057	−0.0101**
			(0.004)	(0.004)
**Age of household head**			0.0002	0.0003
**Religion (‘Traditionalist’ as reference)**			(0.0002)	(0.0002)
Christian				−0.0162
				(0.027)
Muslim				−0.0084
**Ethnicity (‘Others’ as reference)**				(0.027)
Minorities				0.0025
				(0.005)
Fulani				0.0295***
				(0.009)
Yoruba				0.0236***
				(0.007)
Igbo				−0.0162***
				(0.004)
Hausa				−0.0015
				(0.006)
**Obs.**	**10820**	**10820**	**10820**	**10820**

Results are based on average marginal effect from the bivariate probit regression for literacy and under-5 mortality. Robust standard errors in parentheses. rho = −0.14: residual correlation coefficient between under-5 mortality and access to maternal care. Wald test of rho = 0: chi2(1) = 12.5408; p-values of rho’s Wald test = 0.0004. *** p < 0.01, ** p < 0.05, * p < 0.1

**Table 10:** Regression results: literacy and access to maternal care.

Model	(1)	(2)	(3)	(4)
**Literacy (‘Not able to read’ as reference)**				
Able to read	0.0336***	0.04310***	0.0385***	0.0216**
	(0.008)	(0.00934)	(0.009)	(0.010)
**Age (‘15-24’ as reference)**				
25-29	0.0326***	0.0341***	0.0342***	0.0284**
	(0.010)	(0.010)	(0.010)	(0.010)
30-34	0.0591***	0.0615***	0.0608***	0.0535***
	(0.011)	(0.010)	(0.011)	(0.011)
35-39	0.1470***	0.1507***	0.1466***	0.1329***
	(0.013)	(0.013)	(0.013)	(0.0134)
40-49	0.2230***	0.2258***	0.2161***	0.2015***
**Wealth index (‘Poor’ as reference)**	(0.016)	(0.016)	(0.018)	(0.018)
Middle		−0.0051	−0.0102	−0.0276**
		(0.010)	(0.010)	(0.010)
Rich		−0.0257**	−0.0364***	−0.0505***
		(0.012)	(0.013)	(0.013)
**Family size**			−0.0039***	−0.0011
			(0.001)	(0.001)
**Location (urban = 1, rural = 0)**			0.0112	0.0202**
			(0.009)	(0.009)
**Age of household head**			0.0010***	0.0012**
**Religion (‘Traditionalist’ as reference)**			(0.0003)	(0.0004)
Christian				0.0706
				(0.050)
Muslim				−0.0445
**Ethnicity (‘Others’ as reference)**				(0.050)
Minorities				0.0459***
				(0.012)
Fulani				−0.0290
				(0.017)
Yoruba				0.0124
				(0.014)
Igbo				−0.0807***
				(0.012)
Hausa				−0.0109
				(0.016)
**Obs.**	**10820**	**10820**	**10820**	**10820**

Results are based on average marginal effect from the bivariate probit regression for literacy and access to maternal care. Robust standard errors in parentheses. rho = −0.14: residual correlation coefficient between under-5 mortality and access to maternal care. Wald test of rho = 0: chi2(1) = 12.5408; p-values of rho’s Wald test = 0.0004. *** p < 0.01, ** p < 0.05, * p < 0.1

When family size, urban location, and age of household head are added, the marginal effect of literacy barely changes (0.32 ppt to 0.33 ppt) but remains insignificant, while the marginal effect decreases modestly from 4.31 ppt to 3.85 ppt for access to maternal care but still significantly positive. The addition of ethnicity and religion flips the sign from slightly negative to slightly positive, but the marginal effect remains extremely small and statistically non-significant for under-five mortality, and for access to maternal care, the marginal effect of literacy drops substantially from 3.8 ppt to 2.2 ppt but remains statistically significant. This suggests that after adjusting for socioeconomic and sociocultural factors, literacy still has no independent association with child mortality, but for access to maternal care, the marginal effect shrinks but does not disappear.

Tables 11 and 12 respectively present results for the inclusion of both formal education and literacy in the bivariate regression for under-five mortality and access to maternal care. Literacy has no significant association with under-five mortality and access to maternal care. However, the coefficient of secondary education increased from 1.56 ppt to 1.92 ppt for under-five mortality (Table 11). In Table 12, primary, secondary and tertiary education levels remain significantly associated with access to maternal care with slightly increased coefficients (5.32 ppt, 9.45 ppt and 7.54 ppt). On addition of ethnicity and religion, the remaining significance of formal education was further attenuated as both literacy and all education levels became statistically insignificant for under-five mortality, indicating that the earlier observed protective association is largely mediated by deep-rooted sociocultural structures rather than by education or literacy. For access to maternal care (Table 12), primary and secondary education remained significantly positive even after controlling for ethnicity and religion, while literacy again showed no independent association both outcomes.

**Table 11:** Regression results with education & literacy: under-five mortality.

Model	(1)	(2)	(3)	(4)	(5)
**Education (‘No education’ as reference)**					
Primary education	−0.0080	−0.0060	−0.0061	−0.0080	0.0003
	(0.005)	(0.005)	(0.005)	(0.006)	(0.005)
Secondary education	−0.0208***	−0.0151***	−0.0156***	−0.0192***	−0.0054
	(0.004)	(0.004)	(0.005)	(0.006)	(0.006)
Tertiary education	−0.0190***	0.0054	0.0056	−0.0102	0.0058
**Age (‘15-24’ as reference)**	(0.005)	(0.008)	(0.008)	(0.010)	(0.011)
25-29	−0.0143***	−0.0133***	−0.0140***	−0.0143***	−0.0096**
	(0.005)	(0.005)	(0.005)	(0.005)	(0.005)
30-34	−0.0155***	−0.0144***	−0.0154***	−0.0156***	−0.0101**
	(0.005)	(0.005)	(0.005)	(0.005)	(0.005)
35-39	−0.0234***	−0.0219***	−0.0233***	−0.0235***	−0.0162***
	(0.005)	(0.005)	(0.005)	(0.006)	(0.006)
40-49	−0.0086	−0.0070	−0.0098	−0.0098	−0.0036
**Wealth index (‘Poor’ as reference)**	(0.007)	(0.007)	(0.007)	(0.007)	(0.007)
Middle		−0.0021	−0.0013	−0.0018	0.0025
		(0.004)	(0.004)	(0.004)	(0.004)
Rich		−0.0177***	−0.0158***	−0.0164***	−0.0107*
		(0.005)	(0.005)	(0.005)	(0.006)
**Family size**			−0.0005	−0.0005	−0.0011**
			(0.0005)	(0.0005)	(0.0005)
**Location (urban = 1 & rural = 0)**			−0.0057	−0.0058	−0.0101**
			(0.004)	(0.004)	(0.004)
**Age of household head**			0.0003	0.0002	0.0003*
			(0.0002)	(0.0002)	(0.0002)
**Literacy**				0.0056	0.0067
				(0.005)	(0.005)
**Religion (‘Traditionalist’ as reference)**					
Christian					−0.0151
					(0.027)
Islamic					−0.0078
					(0.027)
**Ethnicity (‘Others’ as reference)**					
Minorities					0.0024
					(0.005)
Fulani					0.0289***
					(0.009)
Yoruba					−0.0014
					(0.006)
Igbo					−0.0161***
					(0.004)
Hausa					0.0232***
					(0.007)
**Obs.**	**10820**	**10820**	**10820**	**10820**	**10820**

These results are based on average marginal effect from the bivariate probit regression with education and literacy for under-5 mortality. Robust standard errors in parentheses. rho = −0.14: residual correlation coefficient between under-5 mortality and access to maternal care. Wald test of rho = 0: chi2(1) = 12.1377; p-values of rho’s Wald test = 0.0005. *** p < 0.01, ** p < 0.05, * p < 0.1

**Table 12:** Regression results with education & literacy: access to maternal.

Model	(1)	(2)	(3)	(4)	(5)
**Education (‘No education’ as reference)**					
Primary education	0.0422***	0.0538***	0.0516***	0.0532***	0.0283**
	(0.011)	(0.012)	(0.012)	(0.012)	(0.012)
Secondary education	0.0674***	0.0949***	0.0895***	0.0945***	0.0509***
	(0.009)	(0.011)	(0.012)	(0.014)	(0.014)
Tertiary education	0.0343**	0.0774***	0.0700***	0.0754***	0.0281
**Age (‘15-24’ as reference)**	(0.0013)	(0.017)	(0.017)	(0.020)	(0.019)
25-29	0.0325***	0.0349***	0.0344***	0.0346***	0.0299***
	(0.010)	(0.009)	(0.010)	(0.010)	(0.010)
30-34	0.0595***	0.0627***	0.0609***	0.0610***	0.0555***
	(0.010)	(0.010)	(0.011)	(0.011)	(0.011)
35-39	0.1466***	0.1524***	0.1470***	0.1471***	0.1362***
	(0.013)	(0.013)	(0.013)	(0.013)	(0.013)
40-49	0.2296***	0.2349***	0.2232***	0.2232***	0.2079***
**Wealth index (‘Poor’ as reference)**	(0.017)	(0.016)	(0.018)	(0.018)	(0.018)
Middle		−0.0323***	−0.0356***	−0.0350***	−0.0376***
		(0.010)	(0.011)	(0.011)	(0.010)
Rich		−0.0596***	−0.0661***	−0.0651***	−0.0583***
		(0.014)	(0.014)	(0.014)	(0.014)
**Family size**			−0.0029***	−0.0029***	−0.0009
			(0.001)	(0.001)	(0.001)
**Location (urban = 1 & rural = 0)**			0.0115	0.0117	0.0194**
			(0.009)	(0.009)	(0.009)
**Age of household head**			0.0010**	0.0010***	0.0012***
			(0.0004)	(0.0004)	(0.0004)
**Literacy**				−0.0070	0.0009
				(0.012)	(0.011)
**Religion (‘Traditionalist’ as reference)**					
Christian					0.0599
					(0.050)
Islamic					−0.0458
					(0.050)
**Ethnicity (‘Others’ as reference)**					
					0.0461***
Minorities					(0.012)
					−0.0260
Fulani					(0.017)
					−0.0131
Yoruba					(0.016)
					−0.0816***
Igbo					(0.012)
					0.0144
					(0.014)
**Obs.**	**10820**	**10820**	**10820**	**10820**	**10820**

Results are based on average marginal effect from the bivariate probit regression with education & literacy for access to maternal care. Robust standard errors in parentheses. rho = −0.14: residual correlation coefficient between under-5 mortality and access to maternal care. Wald test of rho = 0: chi2(1) = 12.1377; p-values of rho’s Wald test = 0.0005. *** p < 0.01, ** p < 0.05, * p < 0.1

### 3.4. Robustness checks

A series of robustness checks, including alternative estimation strategies and model specifications, are reported in S1 Appendix. Propensity score matching (PSM) was used to compare mothers with different levels of education to those with no education. The choice of PSM was because it allows the assessment of whether the associations of maternal education with access to maternal care and under-five mortality persist when comparing women with similar socioeconomic and demographic profiles. Propensity scores were estimated using a logistic model that included maternal age, location (urban), wealth index, age of household head, family size, ethnicity, and religion. Nearest-neighbour matching with replacement (1:1) was applied. Covariate balance was assessed using standardized mean differences and variance ratios, following established guidelines that define standardized differences below 10% [37,38 & 2011], and variance ratios between 0.8 and 1.25 as evidence of adequate balance [39,40]. Across all specifications, the matching procedure produced substantial improvements in balance. In the raw sample, several covariates exhibited severe imbalance, with standardised mean differences exceeding 0.50 or more (e.g., wealth index = 1.61; urban residence = 0.54; family size = –0.60; religion = –1.08). After matching, all standardised differences fell below the 10% threshold, ranging from –0.08 to 0.03, indicating that treated and control groups of mothers in the matched sample were well aligned on observable characteristics. Variance ratios also improved considerably. While the raw variance ratios ranged from 0.34 to 1.98, all matched values fell between 0.80 and 1.18, which is within the acceptable range for PSM-based balance diagnostics. These improvements were also visually confirmed through density and boxplots of the propensity score distributions, which showed limited overlap in the unmatched sample but near-complete overlap after matching, demonstrating successful achievement of common support.

Using the matched samples, we estimated the average treatment effect on the treated (ATET) by treating each education category as a separate treatment and comparing mothers at each education level with those who had no education. The average treatment effect on the treated (ATET) for under-five mortality and access to maternal care come under two specifications: adjustment for only maternal age and inclusion of full covariates (maternal age, wealth index, urban location, family size, age of household head, ethnicity and religion).

On one hand, the PSM results show that primary education has no detectable effect under either specification. Secondary and tertiary education are associated with small but statistically significant reductions in under-five mortality in the maternal-age-only model (2.1 ppt and 1.8 ppt, respectively). However, when full covariates are included in Column (2), these associations disappear entirely, with ATET values close to zero and statistically non-significant. This reinforces the conclusion from the bivariate probit model that the apparent protective association of maternal education with child survival is largely explained by underlying socioeconomic, demographic, and sociocultural characteristics rather than by education itself. On the other hand, the PSM estimates confirm a strong and consistent education gradient for access to maternal care. Primary, secondary, and tertiary education are all significantly associated with higher utilisation of maternal healthcare, with secondary education showing the largest improvement (6.7 ppt). After including the full covariates in, the magnitudes decrease but remain statistically significant for primary (2.8 ppt) and secondary education (5.4 ppt), while tertiary education becomes smaller and statistically insignificant. These patterns align closely with the bivariate probit results, which also show that primary and secondary education remain robust predictors of maternal care uptake even after controlling for wealth, ethnicity, religion, and household characteristics.

In addition to PSM, we estimated separate probit models for each outcome. While the bivariate probit model formally accounts for correlated unobserved factors associated with access to maternal care and under-five mortality, the estimated marginal effects are very similar to those obtained from separate probit models. This similarity indicates that the two outcomes share some unobserved factors but not to an extent that substantially alters the single-equation marginal effects. In this context, the primary advantage of the bivariate approach lies in the correct modelling of the joint error structure and the improved efficiency of the estimates, rather than in large changes to the marginal effects themselves.

## 4. Discussion

Our findings show that higher levels of maternal education, particularly at the secondary level, are significantly associated with a lower likelihood of under-five mortality. This is consistent with previous research indicating that children of more educated mothers tend to experience better survival outcomes [19,23,24,26,27]. Several studies suggest that secondary education is linked to health-enhancing behaviours and knowledge, which may contribute to improved child health [41,42]. In Indonesia, Mellington and Cameron [28] found that each additional year of maternal education was associated with reduced child mortality. Similarly, Glewwe [20] in Morocco and Chou et al. [21] in Taiwan found that maternal education was linked to better child-rearing and infant health outcomes, respectively. Education may enhance health literacy, enabling mothers to recognize symptoms early, adhere to treatment, and adopt preventive health practices, although the precise mechanisms may vary across contexts.

The strength of the association between maternal education and under-five mortality declines when economic status is included in the model, suggesting that part of this relationship may be confounded by household wealth. This finding aligns with prior studies [43,9,15], which note that wealthier households typically enjoy better nutrition, living conditions, and access to healthcare services. In particular, the lowest likelihood of under-five mortality is observed among children from the highest wealth quintile, possibly reflecting a threshold effect where only the most affluent households can afford consistent and high-quality healthcare and related resources.

When ethnicity is accounted for, the association between education and under-five mortality becomes statistically insignificant across all education levels, indicating that ethnicity may be a critical contextual factor shaping health outcomes. As noted by [18], ethnic variation in child mortality is substantial. In our sample, Fulani and Yoruba households, largely concentrated in northern and southwestern Nigeria, exhibit higher under-five mortality rates. These groups often face specific challenges, such as nomadic lifestyles, limited access to formal healthcare and education, cultural norms surrounding child-rearing, and exposure to regional instability. In contrast, Igbo households in the south show lower under-five mortality, highlighting the importance of sociocultural and geographic heterogeneity in shaping health outcomes. These patterns suggest that education-based interventions may need to be tailored to reflect the distinct cultural, mobility, and health service contexts of different ethnic groups.

Maternal education is also positively associated with use of maternal healthcare services across all education levels, with secondary education showing the strongest link. This is consistent with a large body of literature that identifies maternal education as a key factor associated with increased utilization of antenatal care, institutional delivery, and postnatal services [44,45,46,47]. For example, Amwonya et al. [47] observed in Uganda that educated mothers were more likely to access comprehensive maternal care, while Nuamah et al. [45] in Ghana found that education enhanced both confidence and awareness in seeking health services. Similarly, Ketema et al. [46] in Ethiopia reported that education was associated with better birth preparedness and complication readiness. In Nigeria, several studies confirm this trend [48,25,49,50,25,51], further highlighting the pivotal role education plays in promoting maternal health-seeking behaviours.

Interestingly, after controlling for economic status, we observe a strengthened association between education and maternal healthcare access, alongside a negative association between wealth and access to maternal healthcare. This finding implies that maternal education may influence healthcare-seeking behaviours independently of economic advantage. A further consideration relates to the counterintuitive finding that wealth is negatively associated with maternal care access once education and other controls are included. There may be several explanations for this finding. First, the DHS/MIS measure of maternal care reflects the utilisation of antenatal and postnatal services typically captured within the formal public health sector. Women from wealthier households may disproportionately rely on private clinics, specialist providers, or informal care arrangements that are not fully captured in the standard DHS service-use indicators, leading to an apparent reduction in measured utilisation. Second, the result may reflect sample composition: rich households represent a relatively small share of the population, and these women may be concentrated in regions or sociocultural groups with distinct care-seeking patterns or preferences that differ from the national trend. Third, it is possible that education mediates much of the behavioural pathway linking socioeconomic status and maternal healthcare use in this context. With the presence of education, the independent contribution of wealth reduces or becomes negative, consistent with the idea that information, empowerment, and health literacy may be more drivers of maternal care uptake than economic resources alone. This finding diverges from studies such as Nuamah et al. [45], which found that women from wealthier households in Ghana had greater healthcare access and were more likely to engage with maternal and child health information.

We also find that older maternal age is positively associated with access to maternal care, consistent with Nuamah et al. [45]. Older women may be more aware of the importance of healthcare during pregnancy or may be prioritized culturally or medically for care due to perceived higher pregnancy-related risks. Conversely, younger mothers, particularly adolescents, may face stigma, fear, or social restrictions that discourage them from seeking maternal care. In many parts of Nigeria, early pregnancies are socially frowned upon, potentially leading to avoidance of public healthcare settings among younger women.

Ethnicity also shapes access to maternal care, with negative association observed across most ethnic groups except for minority populations. These differences may reflect entrenched cultural attitudes that discourage formal healthcare use, reliance on traditional practices, or lack of awareness about the benefits of maternal health services. Even where services are available, cultural resistance to biomedical care can act as a barrier to uptake.

In many Nigerian communities, ethnic and religious norms play a defining role in women’s autonomy, mobility, and decision-making authority. Among Hausa–Fulani populations, norms surrounding female seclusion, reliance on traditional birth attendants, and the prioritisation of male household authority may constrain educated women from independently accessing formal maternal health services. Conversely, among Igbo communities (where female autonomy and market participation are historically higher), education may more readily enhance women’s ability to recognise complications, negotiate healthcare decisions, and utilise antenatal and postnatal care. Regional evidence shows that even educated women may face sociocultural resistance to institutional delivery or postnatal check-ups in northern Nigeria with predominantly Hausa-Fulani ethnic population compared to their southern counterpart [23,18]. These patterns suggest that education alone may be insufficient to overcome entrenched sociocultural barriers in some groups, and that health interventions must integrate culturally tailored strategies that engage community leaders, and address gender norms.

The significant and negative correlation between the error terms of the under-five mortality and maternal care models suggests the presence of unobserved factors that simultaneously influence both outcomes. This shared influence reinforces the notion that improving access to maternal care could be associated with improvements in child survival, even if these relationships are jointly shaped by deeper structural or behavioural characteristics [52]. Such unobserved characteristics may include parental health-seeking attitudes, household preferences for modern medicine, or local health infrastructure, factors that likely associate with both maternal care utilization and child health outcomes.

Lastly, we observe important rural-urban differences in how maternal education is associated with child survival and healthcare access. In contrast to findings from Indonesia, where maternal education was more strongly associated with reduced child mortality in urban areas [28], our study finds stronger associations in rural Nigeria. The stronger association between maternal education and both under-five mortality and maternal care utilisation in rural settings likely reflects the structural and informational constraints that women face outside urban centres. In rural areas, where health facilities are fewer, distances are greater, and transportation infrastructure is limited, educated women may be better able to overcome logistical and informational barriers, interpret health risks, and navigate pathways to appropriate care. The quality and consistency of formal education often differ markedly between rural and urban locations in Nigeria. Rural schooling may be of lower quality which could imply that only women who achieve secondary education acquire meaningful health-related competencies, thereby generating sharper gradients in health outcomes. Urban women, regardless of educational attainment, tend to have closer proximity to facilities, more diverse care options, greater exposure to health information, and stronger social networks that diffuse knowledge about maternal and child health. Education may operate as a stronger differentiator in rural contexts where baseline access is relatively lower, while in urban settings the marginal returns to education on health-seeking behaviours are attenuated by more uniformly accessible services. These patterns suggest that structural barriers, service availability, and sociocultural constraints can magnify the role of education in shaping access to maternal care in rural Nigeria.

Beyond formal education, we examined literacy as a distinct dimension of human capital. When examined separately, literacy and formal education exhibited a broadly similar pattern as both were positively associated with access to maternal care, while their negative associations with under-five mortality weakened substantially as socioeconomic and household controls were introduced. In both cases, wealth index and household factors explained the apparent child survival advantage, whereas the positive association with maternal care remained more robust. However, when literacy and formal education were included together, literacy lost significance for both outcomes and only secondary schooling retained an association with lower under-five mortality, with all education levels associating with higher maternal care uptake. With the inclusion of ethnicity and religion, neither literacy nor education remained significant for child survival, although primary and secondary schooling continued to associate significantly with maternal care use. These findings indicate that literacy contributes to maternal health-seeking behaviour when considered alone, but formal education captures a broader set of human-capital and sociocultural advantages, making it the more durable associating factor in the fully adjusted models.

### 4.1. Policy implications

The findings of this study have important implications for maternal and child health policy in Nigeria. Quantitatively, the policy relevance of education is underscored by the magnitude of the estimated associations. Women with secondary education have roughly a 2 ppt lower probability of experiencing under-five mortality and a 5–9 ppt higher likelihood of accessing maternal care compared to women with no education. These differences are substantial in a context where the national under-five mortality rate remains above 100 deaths per 1,000 live births. Modest increases in secondary school attainment could translate into meaningful improvements in both maternal health service uptake and child survival.

The persistent association between formal education, particularly secondary schooling and improved access to maternal care underscores the need to prioritise universal access to at least secondary education for girls. Policies that expand girls’ completion of primary and secondary schooling, especially in rural areas, can strengthen women’s health-seeking behaviours and improve uptake of essential maternal healthcare services.

The attenuation of the association of education with under-five mortality after controlling for ethnicity and religion suggests that education alone is insufficient to overcome deep-rooted sociocultural barriers. Health interventions must therefore be culturally and contextually tailored. Community-based outreach that engages religious leaders, ethnic associations, and local gatekeepers may be more effective in shifting norms around child health and maternal care, particularly among groups where mortality risks remain high.

Since literacy does not exert an independent association when examined together with education alongside sociocultural factors, we posit that broader schooling is the more strategic policy lever compared to basic reading ability. However, strengthening functional literacy through adult education and community programmes may complement formal schooling and support maternal health engagement.

Furthermore, the finding that wealth does not fully explain disparities in maternal care access indicates that education-based strategies can be cost-effective and sustainable complements to financial interventions. While expanding insurance coverage and reducing out-of-pocket payments remain important, enhancing women’s educational attainment can improve health navigation and decision-making regardless of household economic status.

Policy reforms must address persistent rural–urban inequalities in access to maternal services. Expanding rural health infrastructure, deploying mobile clinics, and integrating culturally sensitive health education initiatives can help ensure that the benefits of female education translate into improved maternal and child health across diverse Nigerian communities.

### 4.2. Study limitations

Although this study emphasises associations rather than causal effects, we recognise that the observed relationships may be affected by several forms of endogeneity. Maternal education may be correlated with unobserved household characteristics such as preferences for health-seeking behaviour, or risk attitudes that also affect child survival and access to maternal care. These unobserved factors could bias the estimated associations even after controlling for wealth, ethnicity, religion, and demographic characteristics. In addition, reverse causality cannot be fully ruled out in cross-sectional data. For instance, previous experiences with child loss or maternal health complications may shape women’s subsequent educational trajectories or awareness, thereby generating feedback between outcomes and educational attainment.

Moreso, while sibling-fixed-effects or within-mother comparisons could be a powerful approach for addressing unobserved family-level confounding and strengthen causal inference, the data structure precludes their use in this study. Because children are sampled rather than fully enumerated, sibling groups are incomplete or unidentifiable for many respondents and relying on the small subset of identifiable sibling clusters would compromise representativeness and reduce analytical power. While the use of a bivariate probit model helps to account for unobserved factors jointly associated with access to maternal care and under-five mortality, it does not eliminate the possibility of omitted-variable bias in the education coefficients. The estimated magnitudes have therefore been interpreted as conditional associations rather than causal effects.

Future research could employ approaches that address such endogeneity concerns and strengthen causal inference. One possibility is the use of instrumental variables (IV), exploiting exogenous variation in education such as historical changes in compulsory schooling laws, educational reforms, or geographical variation in school construction. Another possible direction involves quasi-experimental designs, including regression discontinuity designs, difference-in-differences linked to educational policy implementation, or natural experiments arising from conflict-related disruptions in schooling. Additionally, longitudinal datasets would allow researchers to control for unobserved, time-invariant maternal characteristics that may jointly influence education and health behaviours. These methodological extensions would help identify the causal pathways through which maternal education shapes access to maternal care and child survival in Nigeria.

The study relies on self-reported measures of both access to maternal care and child mortality as captured in the DHS/MIS. Self-reports of antenatal and postnatal care may be affected by recall error, social desirability bias, or misinterpretation of what constitutes ‘care’ across different cultural or regional contexts. Although child survival histories in DHS are generally reliable, misclassification or incomplete reporting is still possible, particularly for neonatal deaths or children who died many years prior to the survey. These sources of measurement error may attenuate estimated associations or introduce noise into the outcomes. The analyses do not account for unobserved variation in the availability or quality of healthcare facilities, which can differ significantly across Nigerian regions and may influence both care-seeking behaviour and child survival independently of maternal characteristics. Such unmeasured structural factors could bias the estimated relationships if they correlate with education or ethnicity. Future studies incorporating facility-level quality measures or geospatial data could better isolate the influence of education from the broader healthcare environment.

A further limitation relates to the fact that, although the bivariate probit model accounts for correlated unobserved factors between under-five mortality and maternal care utilisation, it does not impose a hierarchical relationship between these outcomes. Maternal healthcare use may function as an intermediary pathway through which education influences child survival; however, the cross-sectional nature of the data precludes the identification of such sequential causal mechanisms or the estimation of a recursive model in which access to care directly enters the child mortality equation. Implementing such structural models would require stronger identifying assumptions or exogenous instruments for healthcare utilisation, neither of which are available in the MIS. As such, the results are interpreted as joint associations rather than estimates of a causal hierarchy. Future research using structural or longitudinal methods could more formally investigate the mechanisms by which maternal education translates into improved maternal-care use and subsequent child survival.

## 5. Conclusion

This study highlights the critical role of maternal education, particularly at the secondary level in improving child survival and enhancing access to maternal healthcare in Nigeria. These effects are most pronounced in rural areas, where healthcare infrastructure is weak and educational attainment is low [53,54,55,56,7,57,58,59,60,61,62,63,64,34,65,66]. Our results show that while education is associated with better health outcomes, this association is shaped by economic and cultural contexts, notably wealth, ethnicity, and religion. The findings call for integrated policy strategies that prioritize girls’ education, especially at the secondary level, and simultaneously expand access to culturally sensitive maternal health services. Efforts to reduce under-five mortality must not only address educational barriers but also improve healthcare delivery in underserved regions and communities. Expanding educational opportunities for women and aligning health interventions with sociocultural realities are essential steps toward achieving the SDGs in Nigeria.

## Supporting information

S1 Appendix This appendix reports robustness analyses supporting the main findings, including propensity score matching diagnostics, alternative treatment-effect estimates, separate probit regressions, and comparisons of logit and probit marginal effects. (PDF)
