# Supplementary material for: The association of maternal education with access to maternal care and child mortality in Nigeria: A secondary data regression-based analysis
Source: PLoS One. 2026 Feb 5;21(2):e0337367. doi: 10.1371/journal.pone.0337367 (PMC12875495; doi:10.1371/journal.pone.0337367)
Supplement: S1 Appendix — (PDF) [file pone.0337367.s001.pdf]

S1 Appendix

This appendix presents robustness checks supporting the main findings of the study. The analyses include propensity score matching diagnostics, alternative treatment-effect estimations, separate probit regressions, and comparisons of logit and probit marginal effects. All results are intended to assess the sensitivity of the main findings to alternative estimation strategies and model specifications.

A. Propensity Score Matching Diagnostics

Figure A1. Covariate balance density plot before and after matching.

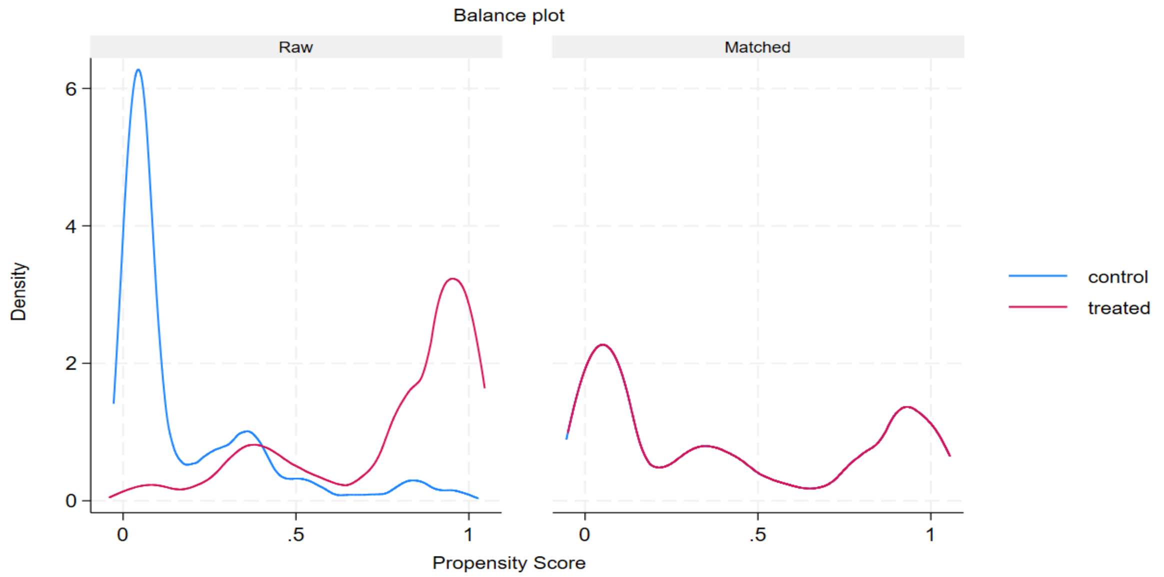

Kernel density plots of propensity scores for treated and control groups before and after propensity score matching. Improved overlap in the matched sample indicates enhanced covariate balance.

Figure A2: Covariate balance box plot before and after matching.

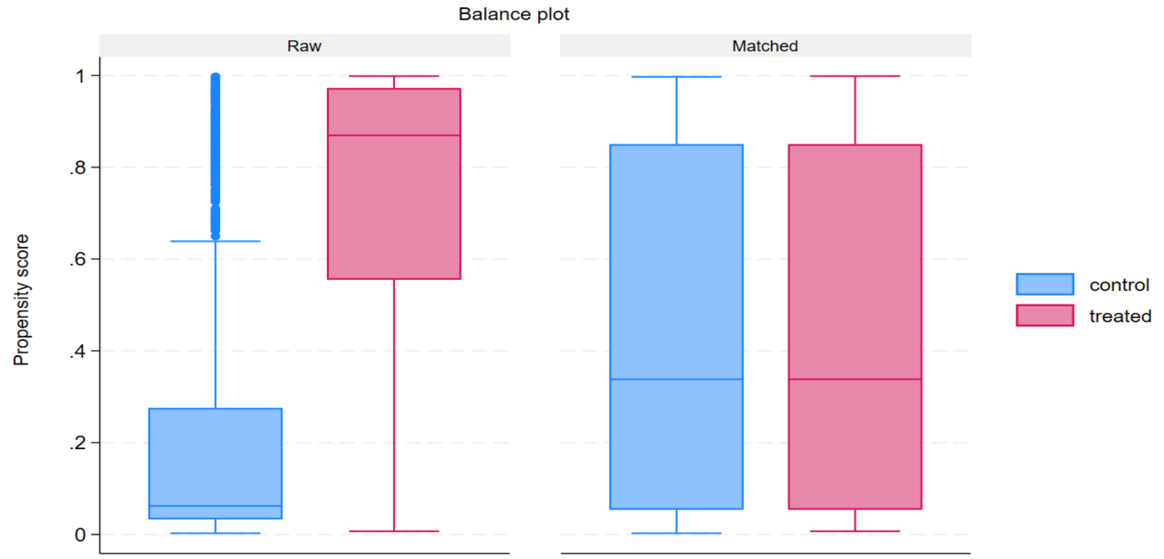

Box plots of standardized mean differences for covariates before and after matching. Values closer to zero after matching indicate improved balance between treated and control groups.

Table A1: Covariate balance summary before and after propensity score matching.

	Standardized	differences	Variance	ratio
	Raw	Matched	Raw	Matched
Age	0.080	-0.062	0.795	0.804
Location (Urban)	0.541	-0.050	1.574	0.958
Wealth index	1.608	0.024	1.394	1.068
Age of HH head	-0.109	0.010	0.942	1.053
Family size	-0.596	-0.003	0.343	1.154
Religion	-1.082	0.032	1.056	0.870
Ethnicity	0.267	-0.076	1.976	1.184
Obs: raw/matched	10,820/21,640			
Treated Obs: raw/matched	4,580/10,820			
Control Obs: raw/matched	6,240/10,820			

Standardized mean differences and variance ratios are reported for unmatched (raw) and matched samples. Values closer to zero for standardized differences and closer to one for variance ratios indicate improved covariate balance.

B. Propensity Score Matching Estimates

Table A2. Average treatment effect on the treated (ATET) from propensity score matching.

	**Under-5 mortality**		**Access to maternal care**	
**Model**	(1)	(2)	(1)	(2)
No education	Ref	Ref	Ref	Ref
Primary	-0.008	0.002	0.043***	0.028**
	(0.005)	(0.007)	(0.012)	(0.013)
Secondary	-0.021***	-0.003	0.067***	0.054***
	(0.004)	(0.004)	(0.009)	(0.011)
Tertiary	-0.018***	0.016	0.039***	0.026
	(0.005)	(0.013)	(0.014)	(0.018)

Notes: Estimated association of maternal education with under-five mortality and access to maternal care, with no education as the reference category. Column (1) includes maternal age only. Column (2) includes the full set of covariates: maternal age, household wealth index, urban residence, family size, age of household head, ethnicity, and religion. Robust standard errors are reported in parentheses. ***p < 0.01, **p < 0.05, *p < 0.1.

C. Alternative Probit Specifications

Table A3. Probit regression marginal effects for under-five mortality.

	(1)	(2)	(3)	(4)
Primary	-0.008	-0.006	-0.006	0.002
	(0.005)	(0.005)	(0.005)	(0.005)
Secondary	-0.020***	-0.015***	-0.015***	-0.001
	(0.004)	(0.005)	(0.005)	(0.005)
Tertiary	-0.019***	-0.005	-0.005	0.012
	(0.005)	(0.008)	(0.009)	(0.011)
25-29	-0.014***	-0.013***	-0.014***	-0.009*
	(0.005)	(0.005)	(0.005)	(0.005)
30-34	-0.015***	-0.014***	-0.015***	-0.010*
	(0.005)	(0.005)	(0.005)	(0.005)
35-39	-0.023***	-0.022***	-0.023***	-0.016***
	(0.005)	(0.005)	(0.006)	(0.006)
40-49	-0.009	-0.007	-0.010	-0.004
	(0.007)	(0.007)	(0.007)	(0.007)
Middle		-0.002	-0.002	0.003
		(0.004)	(0.004)	(0.004)
Rich		-0.018***	-0.016**	-0.010
		(0.005)	(0.006)	(0.006)
Family size			-0.001	-0.001***
			(0.001)	(0.000)
Location (Urban=1, rural=0)			-0.005	-0.010**
			(0.004)	(0.004)
Age of household head			0.000	0.000*
			(0.000)	(0.000)
Minorities				0.003
				(0.005)
Fulani				0.029***
				(0.009)
Yoruba				-0.001
				(0.006)
Igbo				-0.016***
				(0.004)
Hausa				0.024***
				(0.0.006)
Christian				-0.014
				(0.027)
Islamic				-0.007
				(0.027)
Observations	10,820	10,820	10,820	10,820

Notes: Marginal effects from probit models estimated under alternative specifications. Robust standard errors are reported in parentheses. ***p < 0.01, **p < 0.05, *p < 0.1.

Table A4. Probit regression marginal effects for access to maternal care.

	(1)	(2)	(3)	(4)
Primary	0.042***	0.054***	0.052***	0.028**
	(0.011)	(0.012)	(0.012)	(0.012)
Secondary	0.068***	0.095***	0.089***	0.052***
	(0.009)	(0.011)	(0.011)	(0.012)
Tertiary	0.034***	0.078***	0.070***	0.029*
	(0.013)	(0.017)	(0.017)	(0.017)
25-29	0.032***	0.035***	0.034***	0.029***
	(0.009)	(0.009)	(0.009)	(0.009)
30-34	0.059***	0.063***	0.061***	0.056***
	(0.010)	(0.010)	(0.011)	(0.011)
35-39	0.147***	0.152***	0.147***	0.136***
	(0.013)	(0.013)	(0.013)	(0.013)
40-49	0.229***	0.235***	0.223***	0.208***
	(0.017)	(0.017)	(0.017)	(0.017)
Middle		-0.032***	-0.036***	-0.038***
		(0.010)	(0.010)	(0.010)
Rich		-0.059***	-0.067***	-0.059***
		(0.014)	(0.014)	(0.014)
Family size			-0.009***	-0.001
			(0.001)	(0.001)
Location (Urban)			0.011	0.019**
			(0.009)	(0.009)
Age of HH head			0.001***	0.001***
			(0.0003)	(0.000)
Minorities				0.046***
				(0.012)
Fulani				-0.027
				(0.017)
Yoruba				-0.013
				(0.016)
Igbo				-0.082***
				(0.012)
Hausa				0.014
				(0.014)
Christian				0.061
				(0.051)
Islamic				-0.045
				(0.051)
Observations	10,820	10,820	10,820	10,820

Notes: Marginal effects from probit models estimated under alternative specifications. Robust standard errors are reported in parentheses. ***p < 0.01, **p < 0.05, *p < 0.1.

D. Functional form sensitivity

Table A5. Comparison of logit and probit marginal effects.

	Logit (under5 mortality)	Probit (under5 mortality)	Logit (access to maternal care)	Probit (access to maternal care)
Primary	0.0023	0.0024	0.0284**	0.0295**
	(0.005)	(0.005)	(0.012)	(0.012)
Secondary	-0.0004	-0.0009	0.0519***	0.0534***
	(0.005)	(0.005)	(0.012)	(0.012)
Tertiary	0.0125	0.0121	0.0291*	0.0292*
	(0.012)	(0.010)	(0.017)	(0.017)
25-29	-0.0089*	-0.0094**	0.0299***	0.0291***
	(0.005)	(0.005)	(0.010)	(0.010)
30-34	-0.0093*	-0.0099**	0.0556***	0.0543***
	(0.005)	(0.005)	(0.010)	(0.010)
35-39	-0.0160***	-0.0159***	0.1362***	0.1344***
	(0.006)	(0.006)	(0.013)	(0.013)
40-49	-0.0034	-0.0035	0.2079***	0.2062***
	(0.007)	(0.007)	(0.018)	(0.018)
Middle	0.0029	0.0028	-0.0377***	-0.0376***
	(0.004)	(0.004)	(0.010)	(0.010)
Rich	-0.0116**	-0.0101	-0.0585***	-0.0592***
	(0.006)	(0.006)	(0.015)	(0.015)
Family size	-0.0012***	-0.0012***	-0.0009	-0.0009
	(0.001)	(0.001)	(0.001)	(0.001)
Location (Urban)	-0.0104**	-0.0097**	0.0194**	0.0203**
	(0.005)	(0.004)	(0.009)	(0.009)
Age of HH head	0.0003**	0.0003**	0.0011***	0.0011***
	(0.000)	(0.000)	(0.000)	(0.000)
Minorities	0.0025	0.0025	0.0459***	0.0468***
	(0.005)	(0.005)	(0.011)	(0.011)
Fulani	0.0280***	0.0286***	-0.0265	-0.0280
	(0.009)	(0.009)	(0.017)	(0.017)
Yoruba	-0.0012	-0.0014	-0.0133	-0.0132
	(0.006)	(0.006)	(0.015)	(0.015)
Igbo	-0.0161***	-0.0158***	-0.0816***	-0.0769***
	(0.004)	(0.004)	(0.012)	(0.012)
Hausa	0.0231***	0.0235***	0.0142	0.0142
	(0.006)	(0.006)	(0.014)	(0.014)
Christian	-0.0149	-0.0143	0.0611	0.0586
	(0.028)	(0.027)	(0.051)	(0.051)
Islamic	-0.0071	-0.0069	-0.0447	-0.0459
	(0.028)	(0.027)	(0.050)	(0.050)
Observations	10,820	10,820	10,820	10,820

Notes: Marginal effects from logit and probit models for under-five mortality and access to maternal care are reported to assess sensitivity to functional form assumptions. Robust standard errors are reported in parentheses. ***$p < 0.01$, **$p < 0.05$, *$p < 0.1$.
